# Hepatopulmonary Fistula: a life threatening complication of hydatid disease

**DOI:** 10.1186/s13019-015-0311-0

**Published:** 2015-07-29

**Authors:** Mohamed Amirali Gulamhussein, Davide Patrini, Jonathan Pararajasingham, Benjamin Adams, Rajeev Shukla, Dimitrios Velissaris, David Lawrence, Nikolaos Panagiotopoulos

**Affiliations:** 1 Department of Cardiothoracic Surgery, University College London Hospitals (UCLH), 16-18 Westmoreland Street, London, W1G 8PH UK; 2 Division of Internal Medicine, University of Patras, Rio Achaia, Patras, 26504 Greece

**Keywords:** Hydatid disease, Hepatopulmonary fistula, Thoracotomy

## Abstract

Despite extensive infection control measures against parasitic diseases, hydatid disease, caused by Echinococcus granulosus, still occurs in a minor group of our population. If the infection is not treated adequately, it goes on to developing life-threatening complications, one of which is hepatopulmonary fistula. These complications usually warrant early surgical intervention, or else may lead to extensive sepsis and ultimately death. We discuss the case of an elderly female suffering from pulmonary hydatid disease, further complicated by a hepatopulmonary fistula and underwent surgical treatment. This case emphasises the importance of early recognition of pulmonary hydatid disease given its atypical nature of presentation before the disease is further exacerbated by this aggressive complication. Furthermore, it is imperative to incorporate radical surgery as the first-line treatment in established hepatopulmonary fistula, in order to prevent further clinical deterioration and curative outcome.

## Background

Hepatopulmonary fistulae, although rare entities, are a common complication of hydatid or amoebic liver disease. The fistula normally forms through transdiaphragmatic penetration, leading to rupture as a large cyst into the lower lobe of the lung. These fistulae may arise due to other secondary causes as well, such as congenital malformations, penetrating liver trauma, hepatobiliary surgery, biliary obstruction and most importantly infective suppuration. Intrapulmonary rupture of a hepatic hydatid cyst is usually uncommon and the underlying cause is mostly the perforation from the right subphrenic space into the posterior basal segment of the right lower lobe [1]. Other routes of fistula formation involve the presence of an underlying infected biloma where the biliary stasis predisposes to an extensive suppurative process, which leads to rupture and erosion through the diaphragm into the pleural space, bronchus or both [2, 3]. This case emphasises the importance of early recognition of a developing fistula and the use of aggressive surgical treatment at an early stage, which has reduced the associated morbidity and mortality from the sequelae of this disease in majority of the cases.

## Case presentation

A 69-year old Caucasian lady presented to the emergency department with a two day history of haemoptysis, pleuritic chest pain and dyspnoea. She had a productive cough with thick, yellowish sputum, associated with intermittent low-grade pyrexia and mild weight loss over the past few weeks. There were no significant comorbidities, history of recent travel or positive family history. She was a non-smoker.

Physical examination revealed a septic patient, in apparent distress and extremely short of breath. Observations revealed pyrexia, tachycardia, tachypnoea and hypoxia with oxygen saturations of 93 % on air. Blood tests revealed a raised C-reactive protein of 347.2 mg/L along with a raised white cell count of 28.2 × 10^9^ /L. Differential counts revealed neutrophilia 61.7 %, raised lymphocytes 33.2 % and eosinophilia 7 %. All other biochemical tests including urea and electrolytes, liver function tests and clotting profile were within normal range. She was started on broad-spectrum antibiotic therapy and her sputum sample was then cultured which revealed no significant bacterial growth including absence of acid fast bacilli for tuberculosis, however multiple cysts were seen on microscopy. A suspicion of hydatid disease was confirmed by a positive hydatid complement fixation test of the sputum.

Baseline investigations such as an electrocardiogram and echocardiography were unremarkable.

**Fig. 1:**
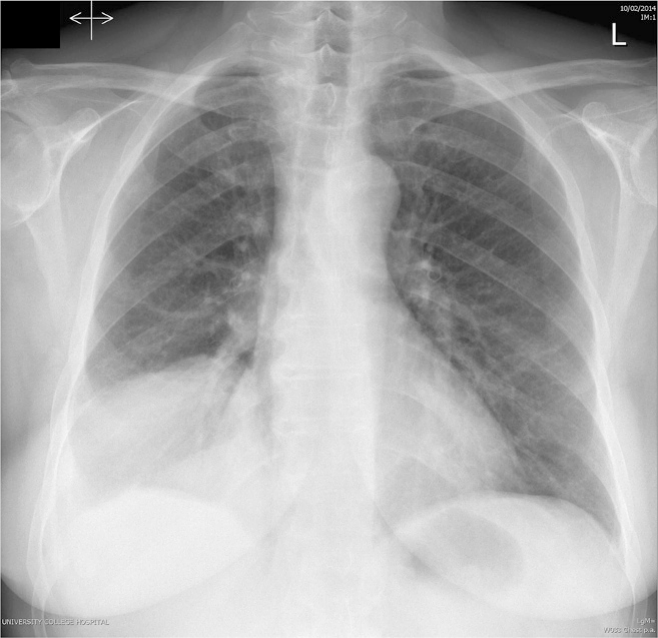
Plain chest radiograph showing a hydropneumothorax on the right side with the pneumothorax component measuring 16 mm. A high right hemidiaphragm is also evident

**Fig. 2:**
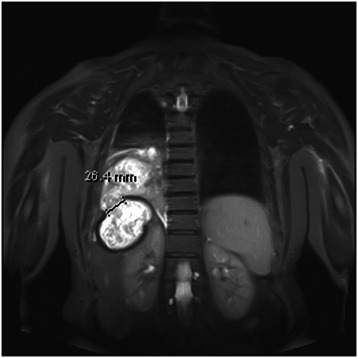
Magnetic resonance imaging scan demonstrating a 26.4 mm fistula in the background of hydatid liver disease. The fistulous communication is shown extending from the right lower lung lobe to the posterior aspect of right liver lobe

A plain chest radiograph showed a hydropneumothorax on the right side with the pneumothorax component measuring 16 mm. The left lung was clear. There was a near-complete whiteout of the right hemithorax with sparing of the apex, which was most likely to be a persistent pneumothorax with possible rupture of the hydatid cyst (Fig. 1). In order to obtain more clear details of the disease process, a magnetic resonance imaging (MRI) scan of the thorax and liver revealed a complex pulmonary mass, which extended from the right lower lobe of the lung, through the hemidiaphragm and into the posterior aspect of the right lobe of the liver. The intrahepatic component appeared thicker walled and well encapsulated. The thoracic component measured 9.5 cm, the intrahepatic component was 9.6 cm and the overall craniocaudal length was 13.2 cm (Fig. 2). There was extensive involvement and rupture of the right posterior diaphragm by the hydatid cyst as it passed into the chest. The mass appeared heterogeneous with a serpiginous internal structure. The chest component appeared more irregular and tubular and was occupying a greater proportion of the lower lobe of the lung, with likely airway involvement. There were further ‘simple’ cysts within the liver and both kidneys. There was a small right pleural effusion, and significantly increased atelectasis in the lower lobe posterior to the hydatid mass.

**Fig. 3:**
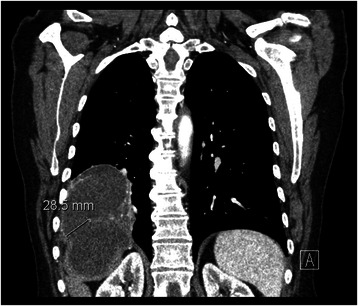
Computed tomography scan demonstrating a complex pulmonary mass excavating anatomical margins and extending into the right hemidiaphragm

The patient was then commenced on the anti-helminthics albendezole and praziquantel. A multidisciplinary team consensus determined that the patient required complex surgical intervention to eradicate the fistula and prevent further deterioration. A computed tomography (CT) scan was performed for accurate surgical planning, delineating the margins to ensure complete division (Fig. 3). The patient underwent a right-sided thoracotomy and a lower lobectomy. There was extensive evidence of thickened pleura with empyema intraoperatively. The fistulous tract was then identified and over sewn. All remaining cystic lesions were removed and curetted and all defects were closed aseptically. Drains were placed post operatively. Following surgery, the patient’s inflammatory markers for sepsis steadily improved. A CT scan post-operatively demonstrated complete resection of the fistula. She continued on the anti-helminthic medications and remained well at six months post discharge.

## Discussion

Hydatid disease is a rare infection known to occur in all age groups. The causative parasite, Echinococcus granulosus, is recognised as having potential to infest any body cavity, organ or tissue. Complications of this infection are rare, though the most common one is hepatopulmonary fistula. In one report, 2 % of the total 1198 cases of hydatid disease had this complication [4]. In some cases, fistula formation is secondary to biliary hypertension as a result of stones blocking the common bile duct [5].

Factors which favour erosion of adjacent structures and subsequent fistula development include the normally high intra-thoracic to intra-abdominal pressure ratio and pericyst formation [6]. A wide range of signs and symptoms normally present prior to fistula development. These include abdominal infection, biliary obstruction in the form of jaundice and respiratory distress. The majority of patients present with symptoms of fever, right upper abdominal pain, chest pain, jaundice and bile-stained sputum [7, 8]. However, the astute clinician should have an index of suspicion when managing for instance right basal pneumonia or infection of the biliary tree, which could mask an underlying advancing fistulous disease. Once the fistula has formed, the patient usually presents with sepsis. There may be bouts of coughing up thick purulent material or bile-containing secretions. Patients usually start expelling cysts in later stages, depending on the size and location of the fistulous tract. There may be associated weight loss and pyrexia, mimicking other chronic infections such as tuberculosis or lung malignancy. Some cases report the development of a hepatopulmonary fistula secondary to radio-ablative treatment of hepatic metastasis [8, 9].

The majority of the cases presented in the literature describe a hepatic presentation of symptoms including jaundice, often with a previous hepatobiliary surgery. Investigations typically yield raised liver enzymes and presence of biliary secretions in sputum or pleural aspirates. Trauma, post-operative biliary stenosis and biliary lithiasis are important predisposing factors. There is little evidence of a pulmonary presentation when the fistula has entered an advanced stage [10, 11].

Conversely, the patient in this case presented with pulmonary symptoms including productive cough with low grade fever, lacking evidence of any hepatobiliary pathology. Past experience of a number of clinicians suggest accurate delineation of the biliary drainage system when considering the causes of this presentation and the management of these cases. The atypical nature of the presentation in this case calls for a wide scope of diagnostic tests when investigating this condition [12, 13].

Radiologically, the presence of a “stack of smoke” appearance on a plain radiograph is suggestive of the development of an underlying fistula. Gold standard investigations include a sinogram or a bronchogram [6]. An important radiological investigation for this condition is contrast enhanced magnetic resonance cholangiopancreatography (MRCP). It not only confirms the diagnosis but also reveals the state of the hepatobiliary tree, which may highlight the underlying cause of the fistula development and helps with surgical planning [8, 12].

The presence of bile in sputum (bilioptysis) or in pleural effusions is pathognomonic of a hepatopulmonary or a biliopulmonary fistula [8]. Eosinophilia and positive hydatid complement fixation test were found in this case, and indeed have been found in a minor number of cases in the past, however radiological means to ascertain the exact cause of the presenting features and manage the advancing process before the patient presents with life threatening systemic sepsis is deemed mandatory in most of the cases [10]. Typically, patients are commenced on concurrent antimicrobial therapy to cover any gram-negative bacteria. Surgical intervention is the second stage in the management and is essential in most cases for definitive treatment [13].

It is vital to keep the pressure low at the fistulous openings, which is normally achieved by thoracostomy tube placement or biliary decompression via endoscopic retrograde cholangopancreatography (ERCP) pre-operatively [7, 14]. In patients with a gross septic picture where multiple abscesses are involved, they are usually drained under CT-guidance. All these methods are subject to change, depending on the patient’s hemodynamic status and the severity of the underlying inflammatory process. In most cases, surgical intervention is warranted and is considered gold standard therapy given the aggressiveness of the disease and the likelihood of rapid deterioration.

Surgical approach via a thoracotomy has become the most widely acquired approach in almost all cases of heptopulmonary fistulae. Surgical steps during thoracotomy include adequate subcostal drainage under direct vision, secured closure of the diaphragmatic perforation, decortication of the affected lobe of lung and lobectomy of the devitalised portion due to the fistulous tract. Other procedures may involve decompression and drainage via endoscopic and interventional radiological means, which together combined, provides an overall favourable clinical outcome. There has been evidence of success via metallic biliary endoprosthesis in the past for similar cases and there is room for incorporating this technique as a viable option of management, which confers less invasiveness [15].

Some studies have indicated spontaneous closure of the fistula (up to 60 % in posttraumatic fistulas) after endoscopic or subcutaneous biliary drainage, while others highlight that conservative means have proven to delay the process of healing, requiring long-standing tedious drainage procedures and introducing new foci of infection into already hemodynamically compromised individuals.

The most challenging step in surgically managing these patients is reconstructing the boundary between the chest and diaphragm, which becomes eroded due to the fistula. Large defects are usually sealed with mobilisation of nearby tissue such as intercostal muscle or pericardial fat, which help restore continuity [8, 13].

Nevertheless all the methods of treatment entirely depend on the gradation of inflammatory markers and patient stability. It is important to note that there is a vast area of development required in terms of ascertaining the most suitable treatment options for this disease. Non-invasive procedures such as endoscopic and bronchoscopic modalities to seal the fistulous communication still remain uncertain as suitable options considering the variable presentation patterns. These methods need to be employed as a treatment algorithm tailored for this disease, as these hybrid approaches could significantly reduce the morbidity and mortality associated with major thoraco-abdominal procedures [10, 14].

## Conclusion

Hepatopulmonary fistulae, although benign in nature, carry an unacceptable mortality risk of up to 10.3 % mainly due to surgical complications [10]. Operative mortality varies from 5 to 50 % and is usually dependent on underlying comorbidities, disease extent and degree of inflammatory process. In some cases, the anaphylactic nature of the organism causes sudden death. A wide spectrum of presentation criteria must be kept in mind whilst identifying this disease. Most patients survive the surgery if operated at an early stage in the disease progression. Current data suggests advocating a combined multidisciplinary approach, involving surgical plus endoscopic and radiological procedures. These modalities have presented as a modern perspective towards the management of this surgical disease. From the first description by Ferguson and Burford in 1967 to the present day, different approaches have been applied and with the introduction of less invasive techniques, the outcomes have significantly improved [10, 11, 14].

## Consent

Written informed consent was obtained from the patient for publication of this case report and any accompanying images.

## Abbreviations

MRI: Magnetic resonance imaging
CT: Computed tomography
MRCP: Magnetic resonance cholangiopancreatography
ERCP: Endoscopic retrograde cholangiopancreatography
